# Nucleolar Proteome Analysis and Proteasomal Activity Assays Reveal a Link between Nucleolus and 26S Proteasome in *A. thaliana*

**DOI:** 10.3389/fpls.2017.01815

**Published:** 2017-10-20

**Authors:** Charlotte Montacié, Nathalie Durut, Alison Opsomer, Denise Palm, Pascale Comella, Claire Picart, Marie-Christine Carpentier, Frederic Pontvianne, Christine Carapito, Enrico Schleiff, Julio Sáez-Vásquez

**Affiliations:** ^1^Laboratoire Génome et Développement des Plantes, Centre National de la Recherche Scientifique, UMR 5096, Perpignan, France; ^2^Laboratoire Génome et Développement des Plantes, University of Perpignan Via Domitia, UMR 5096, Perpignan, France; ^3^Laboratoire de Spectrométrie de Masse BioOrganique, Institut Pluridisciplinaire Hubert Curien, UMR7178 Centre National de la Recherche Scientifique, Université de Strasbourg, Strasbourg, France; ^4^Institute for Molecular Biosciences, Cluster of Excellence Macromolecular Complexes, Buchman Institute for Molecular Life Sciences, Goethe University Frankfurt, Frankfurt, Germany

**Keywords:** proteasome, nucleolus, *Arabidopsis*, nucleolin, FANoS

## Abstract

In all eukaryotic cells, the nucleolus is functionally and structurally linked to rRNA synthesis and ribosome biogenesis. This compartment contains as well factors involved in other cellular activities, but the functional interconnection between non-ribosomal activities and the nucleolus (structure and function) still remains an open question. Here, we report a novel mass spectrometry analysis of isolated nucleoli from *Arabidopsis thaliana* plants using the FANoS (Fluorescence Assisted Nucleolus Sorting) strategy. We identified many ribosome biogenesis factors (RBF) and proteins non-related with ribosome biogenesis, in agreement with the recognized multi-functionality of the nucleolus. Interestingly, we found that 26S proteasome subunits localize in the nucleolus and demonstrated that proteasome activity and nucleolus organization are intimately linked to each other. Proteasome subunits form discrete foci in the disorganized nucleolus of *nuc1.2* plants. *Nuc1.2* protein extracts display reduced proteasome activity *in vitro* compared to WT protein extracts. Remarkably, proteasome activity in *nuc1.2* is similar to proteasome activity in WT plants treated with proteasome inhibitors (MG132 or ALLN). Finally, we show that MG132 treatment induces disruption of nucleolar structures in WT but not in *nuc1.2* plants. Altogether, our data suggest a functional interconnection between nucleolus structure and proteasome activity.

## Introduction

The nucleolus is the most prominent structural and functional nuclear compartment of eukaryotic cells. The main function of the nucleolus is linked with ribosome biogenesis, intimately associated with cell metabolism, proliferation and stress response (Lam et al., 2005; Saez-Vasquez and Medina, 2008; Boulon et al., 2010; Pederson and Powell, 2015). Functional biochemical and proteomic analyses have revealed that the nucleolus is involved in other important biological processes beyond ribosome biogenesis, including RNA metabolism, gene regulation, cell cycle regulation, DNA repair and cell aging (Pendle et al., 2005; Padeken and Heun, 2014; Tsai and Pederson, 2014; Pederson and Powell, 2015; Bensaddek et al., 2016; Palm et al., 2016). Notably the nucleolus plays a role in the cellular response to intrinsic and environmental changes as well as in genome stability and organization (Saez-Vasquez and Medina, 2008; Boulon et al., 2010; Lewinska et al., 2010; Nalabothula et al., 2010; Audas et al., 2012a; Grummt, 2013).

An important property of the nucleolus is that it sequesters a large number of nuclear genes from which RNA polymerases II and III are normally excluded and hence it might play a key role in regulating gene expression (Németh et al., 2010; Németh and Längst, 2011; Padeken and Heun, 2014; Pontvianne et al., 2016b). The nucleolus has also novel and poorly characterized functions in protein sequestering via interaction with other proteins and/or long non-coding RNAs (Audas et al., 2012a,b; Jacob et al., 2012; Lin et al., 2017). The nucleolar retention of specific proteins can potentially suppress or inhibit diverse cellular activities by recruiting general transcription or RNA processing factors or other proteins involved in protein dynamic and activities. Nucleolar sequestering may therefore directly affect post-translational protein modifications and their turnover.

In *Arabidopsis thaliana*, two proteomic studies of the nucleolus have been performed using nucleolar fractions purified from cell cultures (Pendle et al., 2005; Palm et al., 2016). The first analysis identified around 217 proteins in the nucleolus (Pendle et al., 2005). This work revealed several proteins related to the exon-junction complex as well as other non-ribosomal and even “non-nucleolar” proteins. The more recent proteome extends the initial work and identified 1602 proteins in the nucleolar fraction (Palm et al., 2016). Both studies demonstrated also nucleolar localization of spliceosomal proteins and proteins involved in non-sense mediated mRNA decay (NMD) among many others. Splicing factors have been already characterized for their role in the processing of rRNAs (Yoshikawa et al., 2011; Gupta et al., 2014), however, it is not yet known how the nucleolus might impact the activity of spliceosomal or NMD factors.

To have a more precise view of the nucleolar protein content in entire full growing plants, we isolate nucleoli from leave cells by the recently established FANoS (Pontvianne et al., 2013, 2016a). This strategy yielded the identification of most of the factors and complexes involved in rRNA transcription and processing, and in the first steps of ribosome biogenesis. In addition, we identified proteins not observed in the previous approaches using *Arabidopsis* cell cultures (Pendle et al., 2005; Palm et al., 2016). This might be linked to additional nucleolar activities in an entire and growing plant or to cell type specific variations of the nucleolar proteome.

The proteasome is a nuclear-cytoplasmic proteolytic complex involved in nearly all regulatory pathways in eukaryotic cells (Kurepa and Smalle, 2008; Collins and Goldberg, 2017). In particular, the proteasome-ubiquitin system is required for degradation of ribosomal proteins produced in excess (Sung et al., 2016b) or unassembled (Sung et al., 2016a). Furthermore, impaired proteasome function has been correlated with disease in human (Collier et al., 2017; Voutsadakis, 2017) and the stress response in plants (Gladman et al., 2016; Kang et al., 2017; Misas-Villamil et al., 2017). A functional interplay between proteasome and nucleolar activities is in line with the integration of the nucleolus in multiple pathways and its established role as stress sensor (Boulon et al., 2010; Tsai and Pederson, 2014; Pederson and Powell, 2015). Accordingly, we show that nucleolus organization is required for optimal proteasome activity and vice-versa, that inhibition of proteasome activity affects the structure and organization of the nucleolus. The role and biological significance of proteasome subunits in the nucleolus are discussed.

## Results

### Proteomic analysis of the nucleolus

**Figure 1:**
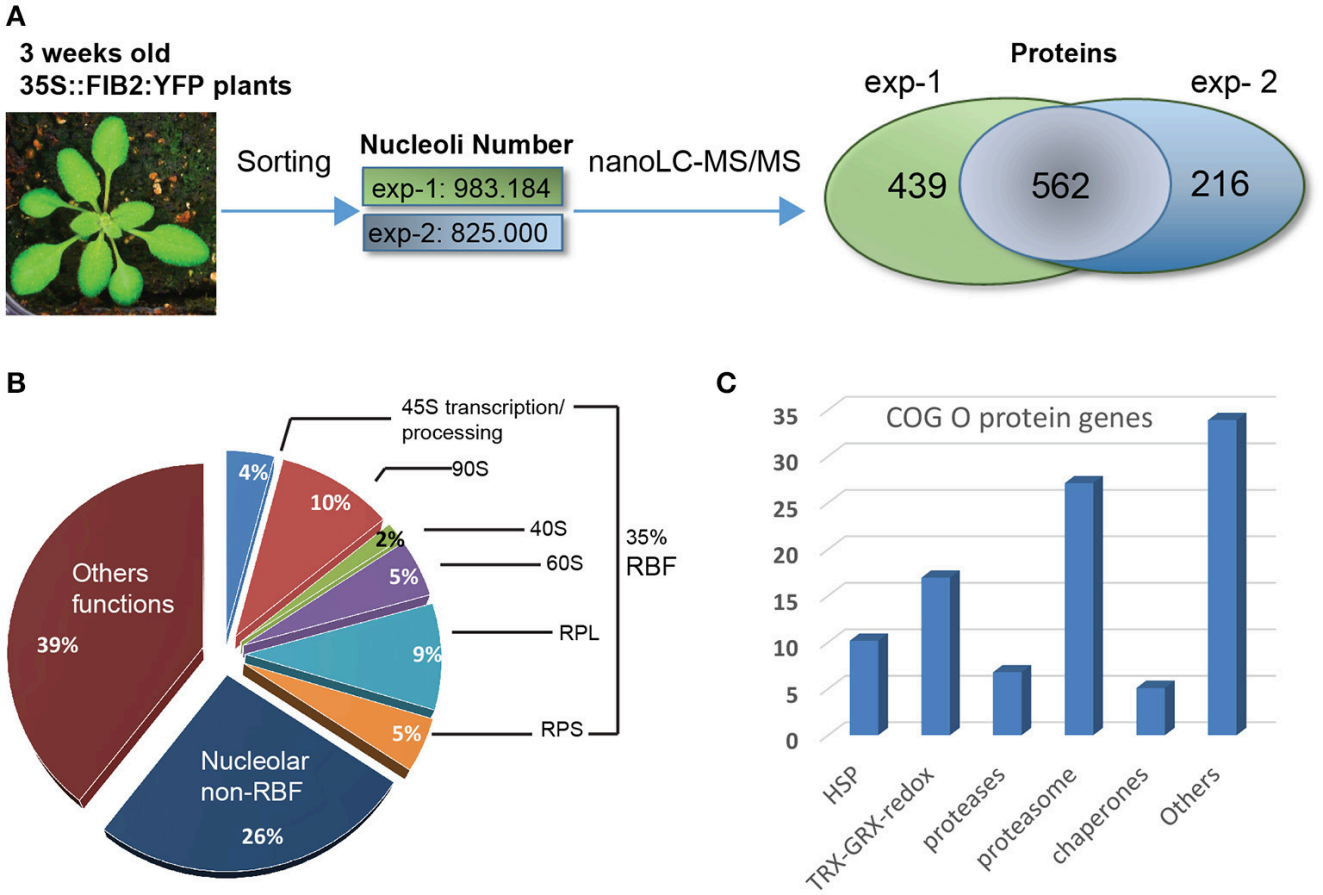
Proteomic analysis of *A. thaliana* nucleoli. **(A)** Nucleoli extraction and nanoLC-MS/MS analysis from biological uplicates: exp-1 and exp-2. From left to right: picture of a 3 week-old leaves from WT FIB2:YFP *A. thaliana*, number of sorted nucleoli per experiment and Venn Diagram showing the number of proteins identified in exp-1 (1,001), exp-2 (778), and both (562). **(B)** Pie graph shows categories of proteins found in the nucleolar fractions. Proteins are classed in three major categories: Ribosome Biogenesis Factors or RBF (35%), nucleolar non-RBF (26%) and others functions (39%). RBF are detailed in sub-categories: 45S transcription/processing (4%), 90S processome (10%), 40S (2%) and 60S (5%) assembly factors, large (9%) and small (5%) ribosome sub-units (RPL and RPS). **(C)** The histogram shows the percentage of nucleolar proteins found in the Cluster of Orthologous Group (COG) O.

We previously reported the FANoS method to purify nucleolar DNA and RNA (Pontvianne et al., 2013, 2016b; Durut et al., 2014). Here, we applied FANoS to investigate the nucleolus proteome of 3 weeks-old *Arabidopsis thaliana* plants. Two nucleoli purifications from leaves of independently grown plants were performed (exp-1 and exp-2). We obtained ~9.83 × 10^5^ and ~8.25 × 10^5^ nucleoli in exp-1 and in exp-2 respectively, and by nanoLC-MS/MS analysis, we identified 1,001 (exp-1) and 778 (exp-2) different proteins (Figure 1A and Tables S1, S2). Comparative analysis revealed that 562 proteins were consistently identified in both experimental data sets (Figure 1A and Table S3). This subset of 562 common proteins identified in both biological replicates was considered in the subsequent analysis, if not otherwise specified. 99 and 409 proteins out of these 562 proteins have been previously identified by Pendle et al. (2005) and Palm et al. (2016) respectively (Figure S1A).

Based on functional characterization and cellular localization studies reported in the literature, we determined that ~35% of the proteins found in nucleolus fractions have been assigned as ribosome biogenesis factors (RBF); including 45S rRNA transcription and processing factors, 90S processome, 40S and 60S assembly factors and ribosomal proteins from large (RPL) and small (RPS) ribosome subunits. ~26% of the 562 proteins have been described as nucleolar proteins, but not yet characterized as RBFs while the remaining 39%, to our knowledge, have not been described as nucleolar and/or having a related nucleolar function (Figure 1B). Then, we assessed in which other subcellular compartments these (562) proteins could be also (transiently or not) localized. We therefore used the subcellular protein distribution report recently published in Palm et al. (2016). We determined that ~75.6% of these proteins have already been reported as nucleolar components with either nucleolar, nucleolar/nuclear or nucleolar/nuclear/cytoplasmic localization revealing the highly proteomic dynamic nature of the nucleolus. ~22.8% were reported as nuclear or nuclear/cytoplasmic and ~1.7% of them were only detected in the cytoplasmic fraction. None of the proteins are localized both in the nucleolus and in the cytosol fractions (Figure S1B and Table S4).

To distinguish particular extra ribosomal biogenesis functions of the nucleolus from *Arabidopsis* leaves, we assessed the enrichment of specific categories of proteins identified in the nucleolar fractions. For that, we compared the 562 protein accessions with a proteomic dataset we obtained from an *Arabidopsis thaliana* whole cell protein extract fraction (Table S5). For comparative purposes, the MS/MS spectrometry analysis of this fraction was performed in a similar manner to that with the nucleolar fractions. A Gene Ontology (GO) analysis revealed that in the nucleolar fractions there is an enrichment of ~3.8X of proteins linked to ribosome biogenesis, ~3.3X of RNA processing factors and ~3.5X of proteins related to Ribo Nucleo Protein (RNP) Complexes compared to whole cell protein extracts (Figure S2A). Additionally, we performed a Cluster of Orthologous Groups (COG) analysis and four major functional categories came up (Figure S2B): COG J (for ribosome biogenesis structure and translation) (17%), COG A (for RNA processing and modifications) (14%), COG O (for post-translational modification, protein turn over and chaperones) (13%) and COG R (for general function prediction only) (12%). These two analyses clearly show that nucleolus of *Arabidopsis* plants is enriched in proteins linked to ribosome biogenesis, to RNA processing and modifications and to RNP complexes. The COG analysis revealed also nucleolar enrichment of proteins linked to protein dynamics. More precisely, in the COG O category, 27% of the proteins correspond to 26S proteasome subunits while the 73% of the remaining proteins include redox activities (17%), HSP (10%), proteases (7%), chaperones (5%), and others (34%) (Figure 1C and Table S6).

All together these results demonstrate that FANoS methodology allows to obtain purified nucleoli from *Arabidopsis* leaves for proteasome analysis. This analysis indicates that in addition to ribosome biogenesis and RNA related factors the nucleolus is enriched in proteins/factors involved in enzymatic reactions and/or gene expression regulation. Because proteasome subunits are the most abundant proteins in the post-translational modification category, protein turn over and chaperones (COG O), we decided to study the functional relevance of the nucleolar localization of these subunits of the 26S proteasome.

### 20S proteasome localization in *Arabidopsis* protoplasts

The proteasome is a sophisticated complex that selectively degrades protein substrates marked by ubiquitin covalent linkage (Kurepa et al., 2009; Liepe et al., 2014; Bach and Hegde, 2016). The 26S proteasome complex is composed of the 19S regulatory and the 20S catalytic subunits. The 19S subunit is organized in two sub-complexes: the lid built of 8 Rpn proteins and the base composed of 3 Rpn proteins (Regulatory Particle Non-ATPase) as well as 6 Rpt (Regulatory Particle Triple-A or Regulatory Particle Triphosphatase) proteins. The 20S subunit is built of 7 alpha and 7 beta proteins (Vierstra, 2003; Kurepa and Smalle, 2008).

Among the 562 proteins identified in our nucleolar fractions, we found components of the lid (Rpn5, Rpn7), the base (Rpt1 and Rpt5), and the alpha (α1, α3-α6) and beta (β3, β5, β7) subunits (Figure S3A, blue labeled). We also detected additional proteins from the lid (Rpn6, Rpn8, Rpn9, and Rpn11), the base (Rpn1 and 2, Rpt2, 3, and 4) and the alpha (α2 and 7) and beta (β1, 2, 4, and 6) subunits in the individual exp-1 or exp-2 data sets (Figure S3A, orange labeled). Accordingly, several of these proteins were also reported in the nucleolus proteome of *Arabidopsis* cells (Palm et al., 2016; Table S7). To verify to which extend proteasome subunits are localized in the nucleolus, we analyzed the subcellular localization of 20S (Rpn5a and Rpt5b) and 19S (PBC1/β3 and PBG1/β7) proteins fused to GFP in *A. thaliana* protoplasts (Figure S3B). We noted that nucleolar localization of Rpn5a and PBG1/β7 is weak and dependent on the N- or C-terminal position of the GFP, while Rpt5b and PBC1/β3 do not show nucleolar localization with none of the constructs, suggesting either that only a small fraction of individual “tagged” proteins are assembled into proteasome and/or that they localized transiently in the nucleolus.

### 20S proteasome localization and activity are altered in *nuc1* mutant plants

Therefore, to investigate a potential functional relationship between nucleolus and 26S proteasome, we determined proteasome localization and activity in *nuc1.2* mutant plants which display a complete structural disorganization of the nucleolus (Pontvianne et al., 2007). *A. thaliana* contains two nucleolin protein genes NUC1 and NUC2, previously named AtNUC-L1 and AtNUC-L2 (Pontvianne et al., 2007, 2010). *nuc1.2* plant corresponds to a T-DNA insertion mutant line described in Pontvianne et al. (2010) and Durut et al. (2014).

**Figure 2:**
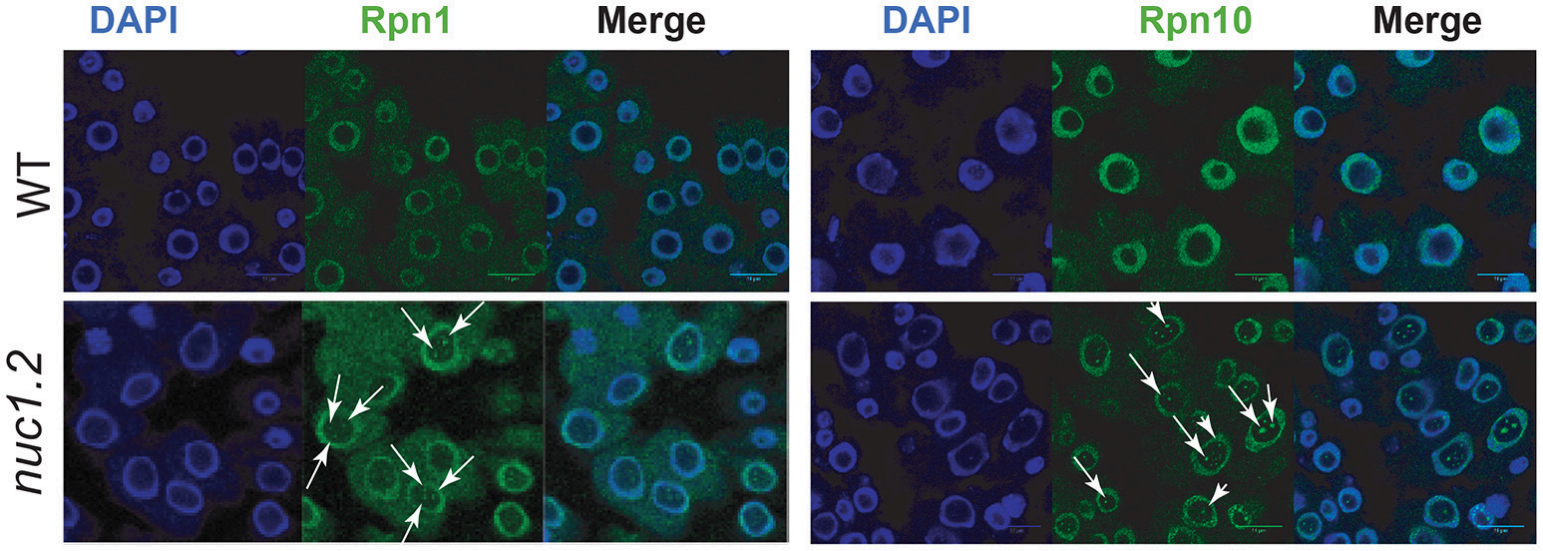
Localization of 26S proteasome subunits in WT and *nuc1.2* plants. Immuno-localization of Rpn1a **(Left)** and Rpn10 **(Right)** proteasome protein subunits in WT and *nuc1.2* root tip cells. Arrows point foci of Rpn1a and Rpn10 proteins in nucleoli of *nuc1.2* mutant cells. DAPI staining was used to visualize nucleoplasm and distinguish the nucleolus.

We first analyzed the cellular localization of proteasome subunits in *nuc1.2* mutant plants (Figure 2). Immunolocalization experiments revealed that, Rpn1 and Rpn10 proteins localize in the cytoplasm and mostly in the nucleoplasm of root apex from WT plants (WT panels). However, Rpn10 might also localize in nucleolar subdomains (Figure S4), which are reminiscent of the nucleolar cavity also called nucleolar vacuoles (Saez-Vasquez and Medina, 2008; Stepinski, 2014). Interestingly, in *nuc1.2* mutants, Rpn1 and Rpn10 proteins form discrete foci (Figure 2, white arrows) in the nucleolus suggesting that localization of proteasome subunits is closely linked to the nucleolus structure.

**Figure 3:**
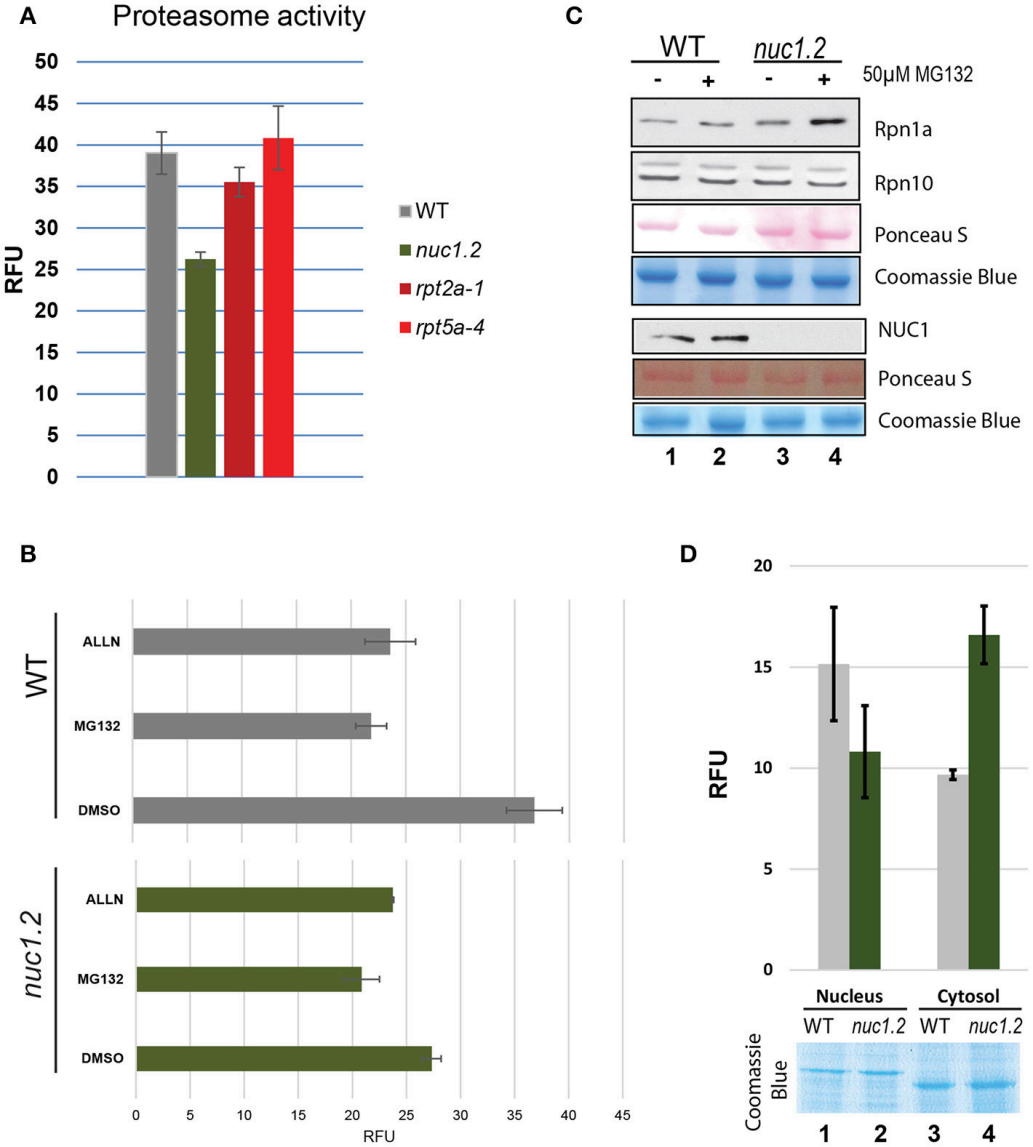
Proteasome 26S activity in plant protein extracts. **(A)** The bar graph shows the proteasome activity in WT (gray), *nuc1.2* (green)*, rpt2-1* (dark red) and *rpt5-4* (red) plant protein extracts. The 20S proteasome activity is shown in Relative Fluorescence Units (RFU). **(B)** Histogram of proteasome activity in WT (gray) and *nuc1.2* (green) protein extracts from plants treated with proteasome inhibitors MG132 or ALLN. Reactions without proteasome inhibitors (DMSO only) were used as control. **(C)** Western blot analysis to determine the protein level of Rpn1a and Rpn10 proteasome subunits in WT and *nuc1.2* mutant plants. Gels and membranes were stained with Coomassie blue or S- Ponceau respectively to verify similar amount of protein in each sample. **(D)** Histogram of proteasome activity in nuclear and cytoplasmic fractions from WT (gray) and *nuc1.2* (green) protein extracts. Gel was stained with Coomassie blue to verify similar amount of protein in each sample. Standard deviation of 3 independent experiments in **(A,B)** and **(D)** is indicated.

Secondly, we performed an *in vitro* 20S proteasome activity assay using total protein extracts from WT and *nuc1.2* plants (Figures 3A,B). The results show that proteasome activity in *nuc1.2* (~26 RFU) is reduced compared to WT (~39 RFU) plants (Figure 3A). Proteasome activity measured in proteasome subunit mutant plants *rpt2* and *rpt5* does not show significant variations (~35 and ~40 RFU, respectively) when compared to WT plant extracts, in agreement with previous reports (Lee et al., 2011; Sakamoto et al., 2011). Then, we investigated 26S proteasome activity in protein extracts from WT and *nuc1.2* plants treated or not with proteasome inhibitors MG132 and ALLN (Figure 3B). WT plants treated with MG132 or ALLN show proteasome activity decrease (~22 RFU for each inhibitor) compared with untreated plants (~37 RFU). Remarkably, in *nuc1.2* mutants, treatment with either MG132 or ALLN inhibitors slightly reduces the proteasome activity (~21 and ~20 RFU, respectively) compared to untreated conditions (~27 RFU). Interestingly, similar proteasome activity is observed between *nuc1.2* (treated or not) and WT treated plants. These results show that *nuc1*.2 is hyposensitive to proteasome inhibitors.

To verify that lower proteasome activity in *nuc1.2* protein extracts is not due to a reduced amount of proteasome protein in *nuc1.2* plants, we determined the amount of two proteasome subunits (Rpn1 and Rpn10) in WT and *nuc1.2* plants, treated or not with the MG132 inhibitor (Figure 3C). Western blot analysis does not reveal significant changes in protein level in WT and *nuc1.2* plants treated or not with MG132, suggesting that proteasome complex amount is not affected in *nuc1.2* plants. Similarly, we checked if NUC1 protein level could be affected by MG132 treatment. However, we do not observe detectable variations of NUC1 protein level in WT plants, suggesting that lower proteasome activity in these plants is not due to changes in the NUC1 protein level after MG132 treatment. If altered post-translational protein modifications of the 26S proteasome complex occur in *nuc1.2* plants, remains to be determined.

Because proteasome activity can be detected both in the nucleus and cytoplasm (Kurepa and Smalle, 2008), we decided to determine proteasome activity in the nuclear and cytoplasmic fractions of *nuc1.2 mutant* plants (Figure 3D). The results show that the proteasome activity is reduced in *nuc1.2* (~10.8 RFU) compared to WT (~15.2) plants in the nuclear fractions, while in cytosolic fractions, proteasome activity is higher in *nuc1.2* (~16.6 RFU) compared to WT (~9.7) plants. Coomassie blue staining shows similar amount of nuclear and cytosolic proteins from *nuc1.2* and WT protein fractions. Detection of H3 histone protein and absence of the cytosolic protein PRXII validates the purity of nuclear fractions (Figure S5).

Altogether these results suggest that functionally structured nucleolus and/or nucleolin protein is required for optimal proteasome dynamics and activity in plants.

### Inhibition of proteasome activity induces nucleolus disruption

It is not known if it is the absence of NUC1 protein or the nucleolar disorganization phenotype observed which is responsible of proteasome localization and/or activity previously observed in *nuc1.2* mutant plants. Thus, we analyzed if inhibition of proteasome activity could have an impact on nucleolus functional organization. Three major structures are visualized in the nucleolus: the Fibrillar Centers (FC), the Dense Fibrillary Component (DFC) and the Granular Component (GC). rDNA transcription localizes to the periphery of the FC, pre-rRNA processing initiates in the DFC and later pre-rRNA processing and ribosome assembly occurs in the GC (Raska et al., 2006; Saez-Vasquez and Medina, 2008).

**Figure 4:**
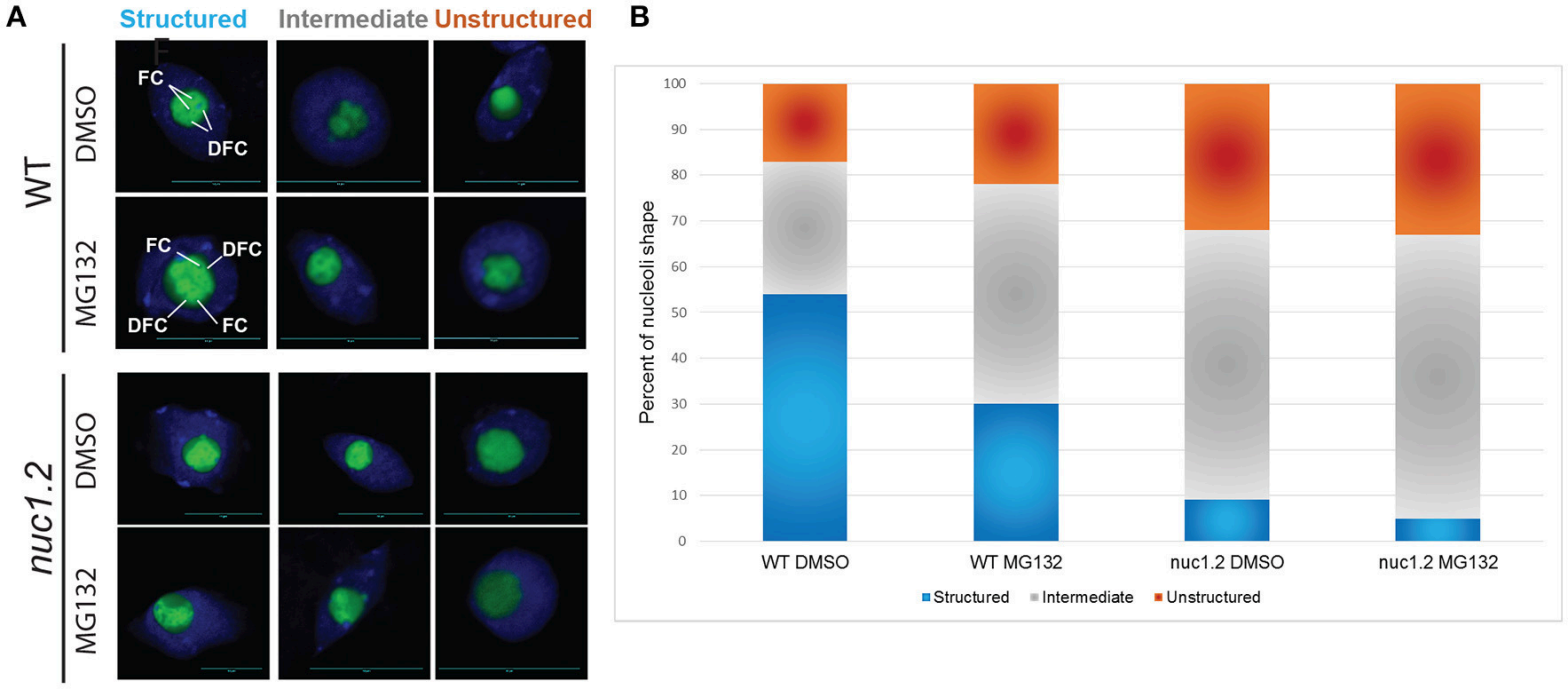
MG132 proteasome inhibitor affects nucleolus structure. **(A)** Nucleoli from WT and *nuc1.2* plants, expressing the Fib2:YFP constructs, treated or not with 50 μM MG132. 100 nucleoli were analyzed in each sample. Green fluorescence of the Fib2:YFP, is used to visualize nucleolus organization with the FC (Fibrillar Centers), and the DFC (Dense Fibrillary Component) components in Structured, Intermediate and Unstructured forms. DAPI staining was used to visualize nucleoplasm and distinguish the nucleolus. **(B)** The bar graph depicts the percentage of structured (blue), intermediate (gray) and unstructured (orange) nucleoli in WT and *nuc1.2* plants treated or not with 50 μg MG132. Reactions without proteasome inhibitors (DMSO only) were used as control. 100 nuclei for each plants and conditions were analyzed. Scale bar = 10 μm.

We investigated nucleolar structure in response to MG132 in WT and *nuc1.2* plants expressing the Fib2:YFP nucleolar marker construct (Fibrillarin2:Yellow Fluorescent Protein) which allows to visualize nucleolus organization through the fluorescence of the YFP (Figure 4 and Picart and Pontvianne, 2017). The green signal of the Fib2:YFP protein reveals 3 distinct states of the nucleolus in WT plants: *Structured*, in which the FC and DFC are clearly recognized, *Unstructured*, in which the FC and the DFC are practically undetectable, and *Intermediate*, where nucleoli cannot be classed in the two previous categories. These three different states are observed in both WT and *nuc1.2* plants treated or not with MG132, although the ratios are clearly different (Figures 4A,B). In untreated WT plants (DMSO only), ~54% of nucleoli appeared to be structured, ~17% are unstructured and ~29% are in an intermediate state (Figure 4B). In contrast, MG132 treatment increases the proportion of unstructured (~22%) and intermediate (~48%) nucleolus states, concomitant with a decreased proportion of structured nucleoli (~30%). In untreated *nuc1.2* mutants (DMSO only), ~9% of the nucleoli are structured, while the others present unstructured (~32%) or intermediate states (~59%), which is in agreement with previous observations (Pontvianne et al., 2007; Picart and Pontvianne, 2017). Remarkably, this analysis shows that MG132 treatment does not result in further unstructured nucleoli in *nuc1.2*, in contrast to WT plants. The fraction of structured, unstructured and intermediate states in *nuc1.2* plants treated with MG132 (~5, ~33, ~62%) remains similar to those observed in untreated plants (~9, ~32, ~59%). This result is also reminiscent to the analysis of proteasome activity in *nuc1.2* showing minimal threshold to MG132 and ALLN (Figure 3B).

Altogether, these data show that inhibition of proteasome activity affects nucleolus structure/organization which might impact on rRNA transcription and processing.

### Proteasome inhibition affects accumulation of pre-RNA in *nuc1.2*

In all eukaryotic cells, nucleolus formation and structure depend essentially on 45S rRNA synthesis and ribosome assembly (Hannan et al., 1998; Grummt, 2003; Sáez-Vásquez and Gadal, 2010). The 45S rRNA genes (encoding 18S, 5.8S, and 25S rRNAs) are transcribed in the nucleolus by RNA polymerase I (RNA pol I) as a single precursor (or pre-rRNA) containing internal (ITS1 and ITS2) and external transcribed spacers (5′ETS and 3′ETS). Pre-rRNA processing depends on the conserved U3 small nucleolar ribonucleoprotein particle (snoRNP) containing fibrillarin and on other transiently associated proteins such as nucleolin (Turner et al., 2009; Phipps et al., 2011; Henras et al., 2015). In *Brassicaceae*, we have shown that the nucleolin-U3 snoRNP complex binds both 5′ETS rDNA and the 5′end of nascent pre-RNA, suggesting coupling of transcription and processing of pre-rRNA (Sáez-Vasquez et al., 2004a,b). Furthermore, we demonstrated that 26S RPN subunits co-purified with the nucleolin-U3snoRNP complex suggesting that 26S proteasome might affect 45S rRNA gene expression (Samaha et al., 2010).

**Figure 5:**
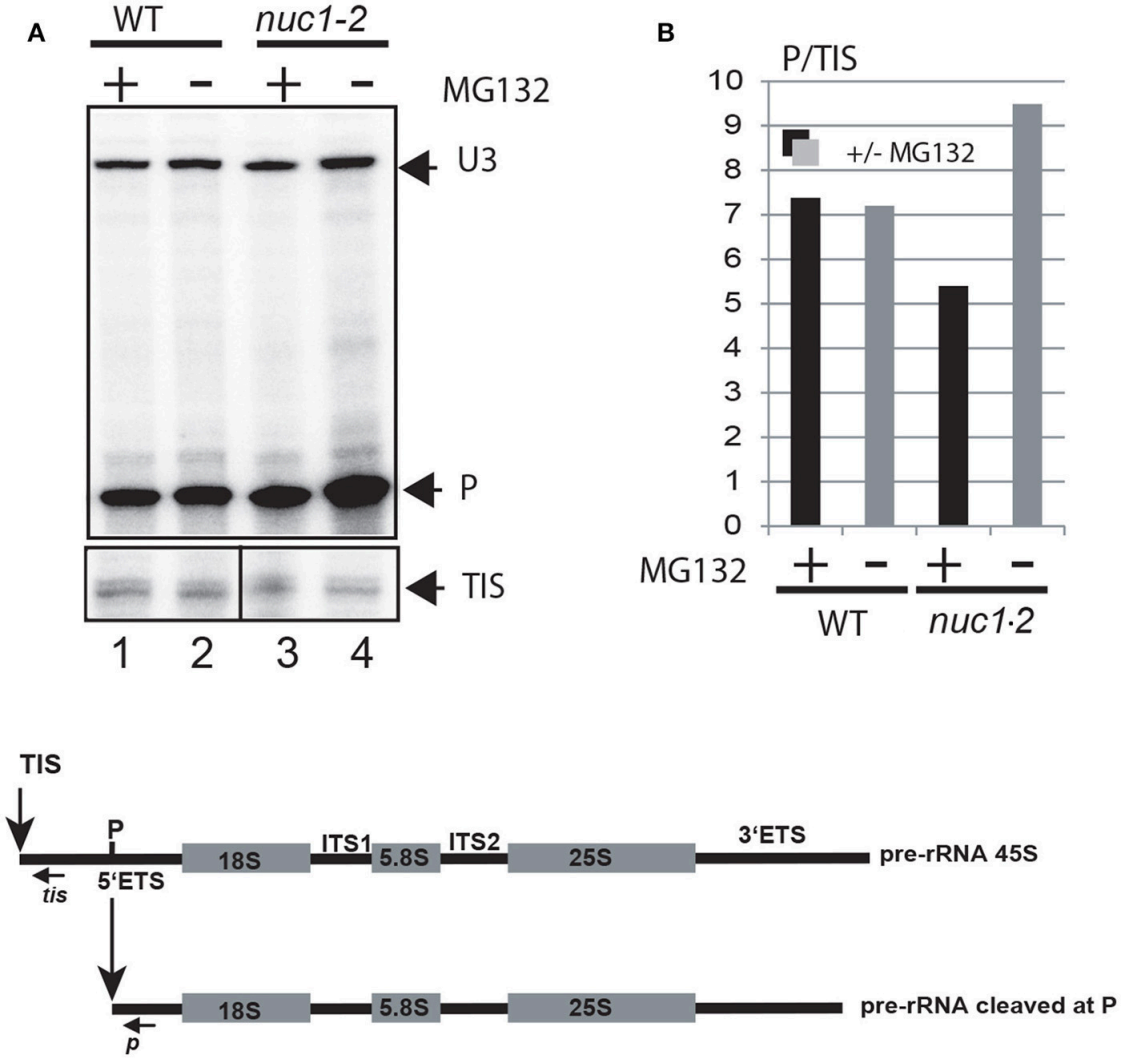
Proteasome inhibitor affects accumulation of pre-rRNA in *nuc1*.2 plants. **(A)** Primer extension experiments were performed using total RNA extracted from WT (lanes 1 and 2) and *nuc1.2* (lanes 3 and 4) plants treated or not with MG132. The relative amount of pre-rRNA initiated at the transcriptional initiation site (TIS) and cleaved at the primary cleavage site (P) was determined using primers *tis* and *p* respectively. Mapping of U3snoRNA (U3) was performed to verify similar amount of RNA in each sample. **(B)** Histogram show ratio of P/TIS signals in each sample. Black and gray bars represent respectively treated and untreated samples with MG132. Below, the scheme shows the 45S pre-rRNAs, containing the external transcribed spacers (5′ETS and 3′ETS), and the structural rRNA sequences (18S, 5.8S, and 25S rRNA in gray boxes) separated by internal transcribed spacers (ITS1 and ITS2). The vertical arrows show the Transcription Initiation Site (TIS) and primary cleavage site (P) in the 5′ETS. Positions of primers used to detect rRNA initiated at the TIS and cleaved at P sites are indicated.

To investigate if 26S proteasomal activity can affect rRNA transcription and or processing, we measured the accumulation of (1) primary pre-rRNA precursor produced by RNA Pol I and (2) processed pre-rRNA at the primary cleavage site (P) in WT and/or *nuc1.2* mutant plants treated or not with MG132 (Figure 5). Primer *tis* maps the transcription initiation site (TIS) (Saez-Vasquez and Pikaard, 1997) while primer *p* maps the P primary cleavage site (Sáez-Vasquez et al., 2004b). To compare the ratio between primary and cleaved pre-rRNA (TIS/P) in WT and *nuc1.2* plants (treated or not with MG132), *tis* and *p* primers were added simultaneously to the same primer extension reactions (lanes 1-4). As previously reported, we observed an accumulation of pre-rRNA precursors in the *nuc1.2* mutant compared to WT plants (Figure 5A, lanes 2 and 4), suggesting a defect in primary pre-rRNA processing in mutant plants (Pontvianne et al., 2007). Interestingly, while P/TIS ratio remains similar in WT plants treated or not with MG132, in *nuc1.2* mutants, P/TIS ratio decreases after MG132 treatment (~5) compared to untreated conditions (~9); signifying an increase of ~2-fold of pre-rRNA cleaved at the P site, or alternatively a decrease of pre-RNA initiated at the TIS, in the *nuc1.2* plants.

Altogether these observations indicate that proteasome inhibition does not affect RNA Pol I transcription or co-transcriptional cleavages of pre-rRNA, but rather later cleavages events taking place in the nucleolus. The data suggest also a role of NUC1 in proteasome activity or complex organization.

## Discussion

We report a proteomic analysis of the nucleolus from *Arabidopsis thaliana* leaves. We identified most of the proteins required for ribosome biogenesis, including rRNA transcription and processing factors, ribosomal proteins and assembly factors, indicating that the FANoS strategy allows the purification of integral nucleoli for nucleolus proteomic analysis (Figure 1 and Tables S1–S4). We also reported ~100 new proteins not identified in previous nucleolar proteome analyses from *Arabidopsis* cell cultures (Pendle et al., 2005; Palm et al., 2016). These novel identified proteins might be cell type, tissue or development specific and they might play a role either in ribosome biogenesis or in other central functions of the nucleolus. Moreover, and in contrast to the previous studies, we obtained the nucleolar proteome from growing plants. Therefore, all signals perceived by the plants are integrated and might impact the nucleolar proteome content, as we know that the nucleolus might also function as a stress sensor (Tsai and Pederson, 2014; Pederson and Powell, 2015).

The nucleolus from *Arabidopsis* leaves contains several factors related to RNA metabolism in the nucleoplasm, in agreement with previous reports in mammalian cells (Ahmad et al., 2009; Bensaddek et al., 2016) and *Arabidopsis* protoplasts (Pendle et al., 2005; Palm et al., 2016). We showed that nucleolus from *Arabidopsis* plants contains also proteins linked to protein metabolism, especially to protein modification and turnover (Figure 1C, Figure S2B and Tables S6, S7). We identified proteasome subunits from the regulatory 20S and the catalytic 19S subunits in the nucleolar fractions, suggesting that 26S proteasome subunits or complexes localize in the nucleolus (Figure 1 and Figures S1–S3). Protein subunits from the 26S proteasome have already been reported in the nucleolus from animal cells (Arabi et al., 2003; Fátyol and Grummt, 2008; Latonen et al., 2011; Jitsukawa et al., 2012; Galimberti et al., 2016), however the functional significance of this localization remains to be completely understood.

Even if most of the subunits of the 26S proteasome complex were identified in the nucleolus from *Arabidopsis* leaves, slight or none nucleolar localization of tested proteasome subunits were observed in roots or mesophyll protoplast nucleoli. Localization of Rpn10 in the nucleolar cavity (NoC) of WT root apical cells (Figure S4) is interesting. The role of these nucleolar subdomains, is not yet clear. NoC are rather characteristic of plants and appear mainly in the actively transcribing nucleoli (Saez-Vasquez and Medina, 2008; Stepinski, 2014). Other proteins showing NoC localization are the AtLa1 protein, demonstrated to bind RNA Pol III primary transcripts (Fleurdépine et al., 2007) and the AtRRP6L1, required for RNA degradation (Lange et al., 2008). In addition, small nuclear and nucleolar RNAs were also shown to localize in this nucleolar subdomain (Shaw and Brown, 2004). It would be then interesting determining more precisely nucleolar localization of the proteasome in leave cells and in other plant tissue and organs.

Nucleolar localization of 26S proteasome might be required for instance for degradation of protein factors involved in transcription and processing of rRNA and/or ribosome assembly. Earlier studies showed a direct role of proteasome in controlling RNA polymerase I transcription and the presence of ubiquitinated pre-rRNA processing factors in the nucleoli in human cells (Stavreva et al., 2006; Fátyol and Grummt, 2008). In plants, 26S proteasome subunits co-purified with the U3snoRNP complex which is required for nucleolar transcription and processing of pre-rRNA (Sáez-Vasquez et al., 2004b; Samaha et al., 2010). In other hand, 26S proteasome dependent degradation of transcriptional regulator c-Myc (Arabi et al., 2003) and protein deubiquitination (Khan et al., 2015; Sun et al., 2015) in the nucleolus of mammalian cells suggest that ubiquitination/deubiquitination might regulate activity of the proteasome in the nucleolus. We cannot exclude neither the possibility that proteasome subunits in the nucleolus might also have activities non-related to proteolyse function, including transcription, DNA repair or chromatin remodeling (Tanaka, 2009).

Our results also indicate that NUC1 protein and/or a structured nucleolus is required for optimal 26S proteasome activity (Figure 3). Remarkably, similar 20S proteasome activities are observed between WT plants treated with proteasome inhibitors and *nuc1.2* mutants. We do not know yet why proteasome activity is reduced in *nuc1.2* protein extracts, nonetheless this is not due to a deregulation of proteasome gene expression, because the level of proteins encoding proteasome subunits is not affected in *nuc1.2* plants (Figure 3C). Interestingly, we observed that the proteasome activity is lower in the nucleus, while it is higher in the cytoplasm of *nuc1.2* plants compared to the WT (Figure 3D). One explanation for these results could be that a putative factor might control negatively proteasome activity or assembly. In this case, in WT plants, this factor should be present in the cytoplasm and down regulates proteasomal activity. In *nuc1.2* mutant, this factor might move to the nucleus, together with the proteasomal subunits (Figure 2), to reduce the proteasomal activity in the nucleus with a concomitant increase in the cytoplasm. Because in whole cell extracts, proteins from nucleus and cytosol are together in a single fraction, the potential proteasome inhibitor factor could globally reduce proteasome activity. This hypothesis is in agreement with a study reporting the involvement of a proteasome inhibitor protein (PAAF1) controlling assembly/disassembly of proteasome in Hela cells (Park et al., 2005). Nevertheless, the involvement of a similar factor and its potential role on proteasome activity in plants remains to be further investigated.

In contrast, we demonstrated that proteasome subunits form discrete foci in the nucleolus of *nuc1.2* plants (Figure 2). Formation of these proteasome-foci might probably affect 26S proteasome assembly or potential modifications required for optimal proteasome activity. The nucleolus is involved in the confinement of nuclear proteins through interactions with long non-coding RNAs (Audas et al., 2012a) or with the ribosomal protein pNON40 (Lin et al., 2017). Thus, it is reasonable to consider that nucleolus might be involved in the regulation of proteasome assembly or activity through a RNA or protein dependent nucleolar sequestering mechanism. Likewise, we cannot exclude that reduced 26S proteasome activity observed in *nuc1.2* protein extracts is due to the absence of NUC1 protein in these plants. Indeed, NUC1 co-purifies with an affinity purified 26S proteasome complex from *Arabidopsis* plants (Sako et al., 2014), while in Hela cells nucleolin might also regulate ubiquitination/deubiquitination status of proteasome targets in response to DNA damage (Lim et al., 2015).

In mammalian cells, proteasome inhibitors induce accumulation of proteasome subunits in the nucleolus (Mattsson et al., 2001; Arabi et al., 2003), nucleolar aggregation of proteasome targets and polyadenylated RNAs (Latonen et al., 2011), increase considerably oocytes nucleolus diameter (Jitsukawa et al., 2012) and accumulation of the stress-inducible transcription factor ATF4 in the DFC and Granular Component (GC) (Galimberti et al., 2016). We showed that inhibition of 26S proteasome activity has a major impact on nucleolus organization, which is reminiscent to the nucleolus disorganization observed in *nuc1.2* mutants (Figure 4 and Pontvianne et al., 2007) and clearly linking proteasome activity with the nucleolar localization of proteasome subunits and functional structures of the nucleolus. Interestingly, inhibition of proteasome activity did not induce significant changes in the accumulation of 45S pre-RNA precursors transcribed by RNA pol I and/or cleaved at the P site in WT plants. In contrast, inhibition of proteasome activity in *nuc1.2* plant mutants affects these primary events. Then, it is reasonable to suggest a fonctional interaction of the 26S proteasome with NUC1 protein activities, since proteasome subunits and nucleolin co-purified with the nucleolin-U3snoRNP complex (Samaha et al., 2010). It would be interesting to investigate how 26S proteasome activity could affect rRNA transcription and processing and more generally ribosome biogenesis to better explain the altered FC and DFC organization observed in WT after proteasome inhibition (Figure 4) but also under different cellular and environmental conditions that might disrupt nucleolus organization.

To conclude, we propose that nucleolar localization of the 26S proteasome is intimately linked to nucleolar activity that is connected with protein synthesis, cell growth and proliferation. Thus, we suggest that 26S proteasome localizes in the nucleolus to control ribosome biogenesis and maybe other cellular processes associated with the nucleolar functions. Nucleolar transit (of 20S or 19S particles or individual protein subunits) might be also required for specific post-translational protein modifications and hence for regulation of proteasome activity (Yedidi et al., 2016). Indeed, the regulation of 26S proteasome activity involves different mechanisms, including post-translational modifications, substitution of catalytic subunits, binding of regulatory complexes and proteasome conformational modifications (Kurepa and Smalle, 2008; Liepe et al., 2014). Investigating, the proteasome nucleolar retention mechanisms, potential proteasome modifications in the nucleolus and how the proteasome might regulate nucleolar functions should be the next steps to better understand the functional link between the nucleolus and proteasome in plants.

## Materials and methods

### Plant materials and growth conditions

All lines were derived from *Arabidopsis thaliana* Columbia (Col 0) ecotype. Plants expressing Fib2:YFP nucleolar marker construct were described in Pontvianne et al. (2013) and Picart and Pontvianne (2017). The *nuc1.2, rpt2a-1*, and *rpt5a-4* T-DNA insertion mutant lines were reported previously in Pontvianne et al. (2007, 2010), Wang et al. (2009), Sakamoto et al. (2011). Seeds were sown either on soil or on 1X Murashige and Skoog medium (MS containing 1% sucrose) and left for 2 days at 4°C to synchronize. Plants were then grown in controlled growth chambers under a 16 h light/8 h dark cycle at 21°C for 3 weeks (FANoS) or 2 weeks *(in vitro* activity assay) or under continuous light for 14 days (Western blot). For treatment with proteasome inhibitors MG132 and ALLN (Sigma), 15-days-old plant seedlings were transferred to petri dishes containing 6 mL of liquid MS medium complemented with 50 μM MG132 and/or 50 μM ALLN for 24 h before harvesting.

### purification of nucleolus by FANoS

Leaves (without petiole) from 3-weeks-old Fib2-YFP plants, were fixed for 20 min in 4% formaldehyde in cold Tris buffer (10 mM Tris-HCl pH7,5, 10 mM EDTA, 100 mM NaCl), and washed twice for 10 min with Tris buffer. Then, leaves were chopped with a razor blade in FACS buffer (45 mM MgCl2, 20 mM MOPS pH7, 30 mM Sodium citrate, 0.1% TritonX-100) containing protease inhibitors (cOmplete, Mini Protease Inhibitor Cocktail from Roche) and filtered through a 30 μM PARTEC CellTrics membrane. Filtrates were sonicated using a Bioruptor (Diagenode) with parameters setted up as follows: three 5-min pulse ON/30-s OFF at Medium intensity. Samples were kept on ice and protected from light until sorting experiment was performed using BD FACS ARIA II (Biosciences), at the IGMM institute, Montpellier (MRI platform), and with parameters described previously in Pontvianne et al. (2016a).

### Nuclear and cytosolic cellular fractionation

Fifteen-days-old plant seedlings were collected, shock-frozen in liquid nitrogen and grinded in fine powder. Samples were homogenized in three volumes of Extraction buffer (20 mM HEPES/KOH, 10 mM MgCL2, 0.5M Hexylene glycol), filtrated through a Miracloth (EMD Millipore Corporation) and a bolting cloth (Sefar AG, 31 μm). Then, Triton X-100 was added (0.5% final) and samples incubated on a rotor for 15 min at 4°C. To obtain cytosolic and nuclear fractions, whole cell extract samples were centrifuged at 1,000 g during 10 min at 4°C. The supernatant corresponds to the cytosolic fraction. The pellet was washed with 1 mL of Extraction buffer containing 0.5% Triton X-100, centrifuged at 1,000 g for 10 min at 4°C and finally resuspended in 150 μL of Extraction buffer (supplemented with 0.5% Triton X-100). This corresponds to the nuclear fraction. For each proteasome activity assay, 1 μg of protein of each fraction was used.

### Liquid chromatography tandem mass spectrometry (LC-MS/MS) analysis

#### Electrophoresis and in gel trypsin digestion

Purified nucleoli fractions were resuspended in Laemmli buffer containing Tris HCL pH 6.8, EDTA 1 mM, 5% of βmercaptophenol, 5% of SDS and protease inhibitors before being separated on an in-house poured 4–10% acrylamide gel. Gel was stained with Coomassie Blue and the lanes were manually cut into six bands of similar size each. Proteins in the gel slices were then reduced, alkylated and digested overnight at 37°C with modified trypsin in a 1:100 enzyme:protein ratio (Promega, Madison, USA). Peptides were extracted during 45 min with 100 μL of 60% acetonitrile, 0.1% formic acid and 15 min with a solution of 100% acetonitrile.

#### Liquid chromatography-tandem mass spectrometry (LC-MS/MS) analyses

LC-MS/MS analyses of nucleoli peptide extracts were performed on a NanoAcquity LC-system (Waters, Milford, MA, USA) coupled to a Q-Exactive plus Orbitrap (Thermo Fisher Scientific, Waltham, MA, USA) mass spectrometer equipped with a nanoelectrospray ion source. Mobile phase A (99.9% water and 0.1% FA) and mobile phase B (99.9% acetonitrile and 0.1% FA) were delivered at 450 nL/min. Samples were loaded into a Symmetry C18 precolumn (0.18 × 20 mm, 5 μm particle size, Waters) over 3 min in 1% buffer B at a flow rate of 5 μL/min. This step was followed by reverse-phase separation at a flow rate of 450 nL/min using an ACQUITY UPLC® BEH130 C18 separation column (200 mm × 75 μm id, 1.7 μm particle size, Waters). Peptides were eluted using a gradient from 1 to 8% B in 2 min, from 8 to 35% B in 43 min, from 35 to 90% B in 1 min, maintained at 90% B for 5 min and the column was reconditioned at 1% B for 20 min.

The Q-Exactive plus Orbitrap instrument was operated in data dependent acquisition mode by automatically switching between full MS and consecutive MS/MS acquisitions. Survey full scan MS spectra (mass range 300–1,800) were acquired with a resolution of 70,000 at 200 m/z with an automatic gain control (AGC) fixed at 3 × 106 ions and a maximum injection time set at 50 ms. The 10 most intense peptide ions in each survey scan with a charge state ≥2 were selected for MS/MS fragmentation. MS/MS scans were performed at 17,500 resolution at 200 m/z with a fixed first mass at 100 m/z, AGC was fixed at 1 × 105 and the maximum injection time was set to 100 ms. Peptides were fragmented by higher-energy collisional dissociation (HCD) with a normalized collision energy set to 27. Peaks selected for fragmentation were automatically put on a dynamic exclusion list for 60 s and peptide match selection was turned on. MS data were saved in.raw file format (Thermo Fisher Scientific) using XCalibur.

#### LC-MS/MS data interpretation and validation

Raw files were converted to.mgf peaklists using msconvert and were submitted to Mascot database searches (version 2.5.1, MatrixScience, London, UK) against an *Arabidopsis thaliana* protein sequences database downloaded from The Arabidopsis Information Resource TAIR site (TAIR10 version), common contaminants and decoy sequences were added. The concatenated database contains 70994 protein entries. Spectra were searched with a mass tolerance of 5 ppm in MS mode and 0.07 Da in MS/MS mode. One trypsin missed cleavage was tolerated. Carbamidomethylation of cysteine residues and oxidation of methionine residues were set as variable modifications. Identification results were imported into Proline software (http://proline.profiproteomics.fr/) for validation. Peptide Spectrum Matches (PSM) with pretty rank equal to one, with peptide length equal to or above seven amino acids and with a Mascot ion score above 25 were kept. False Discovery Rate was then optimized to be below 1% at PSM level.

### Cloning of 26S proteasome subunits and subcellular localization in *Arabidopsis* protoplasts

Subcellular localization was performed as described in Sommer et al. (2011) and Palm et al. (2016). In brief, the coding sequence of Rpn5a (At5g09900), Rpt5b (At1g09100), PBC1/β3 (At1g21720), and PBG1/β7 (At1g56450) genes was amplified using *Arabidopsis thaliana* cDNA and specific oligonucleotides (Table S8). Then, amplified fragments were cloned in the pRTds vector to generate C- and N-terminal GFP fusion constructs. As a nucleolar localization control, atFIB2 (At4g25630) was cloned in front of mCherry into the same vector and co-transformed with the GFP-fusion constructs (Missbach et al., 2013).

Leaves of 4-weeks-old *Arabidopsis thaliana* plants were rubbed on K240 sandpaper and then incubated in 25 mL of extraction buffer [1% (w/v) cellulase R10, 0.3% (w/v) macerozyme in MCP (29 mM MES-KOH pH 5.6, 500 mM sorbitol, 1 mM CaCl2)] for 2 h at 30°C to isolate protoplasts from mesophyll cells. After incubation, the released protoplasts were filtered through a 75 μm nylon mesh and underlayed with 2.5 mL of 100% (v/v) Percoll MCP (pH 5.6 containing 5 mM MES, 500 mM sorbitol, 1 mM CaCl2). After centrifugation at 405 g for 8 min, the clear supernatant of around 20 mL was removed and the remaining protoplast fraction was mixed with the Percoll cushion, followed by overlaying with 7.5 mL 25% (v/v) Percoll in MCP and 5 mL MCP. The mixture was centrifuged at 270 g for 8 min and the green protoplast fraction between MCP and 25% (v/v) Percoll was collected in a new tube. After centrifugation at 100 g for 5 min, the protoplast pellet was diluted in MMg (5 mM MES-KOH pH 5.6, 400 mM sorbitol, 15 mM MgCl2) to a cell number of 106 cells per mL. For transfection, 100 μL protoplasts were mixed with 10 μg pDNA per construct. 100 μL PEG-solution [40% (w/v) PEG-4000, 100 mM Ca(NO3)2, 400 mM sorbitol] was added to the protoplasts. After incubation for 20 min at room temperature, the reaction was stopped with K3-solution (20 mM MES-KOH pH 5.6, 400 mM sucrose, 1 mM CaCl2, MS salts). The protoplasts were incubated over night at room temperature and under constant light condition. The expression analysis was done by confocal laser scanning microscopy (CLSM) using a HCX PL APO CS 40 × 1.25 NA 1.25 oil objective. Transformed protoplasts (around 10 μL) were spotted on an object slide. Fluorescence was excited and detected as follows: GFP 488 nm/505–525 nm, mCherry 568 nm/580–610 nm, chlorophyll fluorescence 514 nm/650–750 nm.

### Proteasome activity assays

Fifteen-days-old plant seedlings treated or not with MG132 or ALLN were collected, shock-frozen in liquid nitrogen and grinded in fine powder. Samples were incubated on ice in Extraction buffer (50 mM HEPES/KOH, 2 mM MgCl2, 150 mM NaCl, 10% Glycerol, 1% Triton X-100) for 30 min with vortexing steps every 10 min. Then samples were centrifuged at 22,000 g for 20 min at 4°C and supernatant recovered. Activity assay was performed using the kit “20S Proteasome Activity Assay Kit” (Chemicon® International) according to the manufacturer's instructions. For each assay, 2 μg of protein extract was used. Fluorescence was determined using “Fluoroskan Ascent FL” (Thermo Scientific), with light excitation at 355 nm and emission at 460 nm.

### Western blot

Plant material (100 mg) treated or not with MG132, was homogenized and extracted in protein extraction buffer [50 mM Tris-HCl pH8, 150 mM NaCl, 10 mM EDTA, 50 mM NaFluoride, 1% NP40, 0.5% Deoxycholate, 0.1% SDS and protease inhibitors (cOmplete, Mini Protease Inhibitor Cocktail from Roche)]. Samples were cleared by centrifugation at 13,000 g for 20 min at 4°C and proteins extracted in 1X SDS-Laemmli buffer. Western blot was performed as described previously (Durut et al., 2014) using, α-H3 (CT, pan from Millipore) α-NUC1 (Pontvianne et al., 2010), α-RPN1a (Wang et al., 2009), α-RPN10 (Lin et al., 2011), and α-PRXII (Bréhélin et al., 2003) antibodies.

### Cytology analysis

Immunofluorescence was performed on roots apex from 8 day –old seedlings as previously described in Durut et al. (2014). Briefly, treated roots were incubated overnight at 4°C with α-RPN1 (1:1,000) and α-RPN10 (1:1,000) and then with anti-rabbit coupled with Alexa 488 (1:1,000, Invitrogen), for 3 0h at room temperature. Slides were then mounted in Vectashield medium containing DAPI solution. For nucleolus structural studies, 2 week-old Fib2-YFP plants (WT and *nuc1.2*, treated or not with MG132), grown on MS medium, were fixed for 20 min in 4% formaldehyde in cold Tris buffer (10 mM Tris-HCl pH7,5, 10 mM EDTA, 100 mM NaCl) and washed twice for 10 min with Tris buffer. Then plants were chopped with a razor blade in LB01 buffer (15 mM Tris-HCl pH 7,5, 2 mM NaEDTA, 0.5 mM spermine, 80 mM KCl, 20 mM NaCl, 0.1% Triton X-100) and filtered through a 30 μM PARTEC CellTrics membrane. Filtrates were completed with an equal volume of sorting buffer (100 mM Tris-HCl pH7,5, 50 mM KCl, 2 mM MgCl2, 0.05% Tween-20, 5% sucrose, filtered through 0.45 μm filter) before spreading on a polysine slide. After air-drying, slides were post-fixed in 2% formaldehyde in phosphate buffer (PBS) for 5 min and washed twice with 1X PBS. Slides were mounted in Vectashield medium containing DAPI solution. Observations and imaging were performed using a confocal microscope LSM 700 from Zeiss.

### Primer extension

Total RNAs from *A. thaliana* WT and *nuc1.2* plant mutants were extracted using Trizol reagent (Invitrogen), according to manufacturer's instructions. Then, all samples were then treated with RQ-DNase (Promega) to eliminate contaminant genomic DNA. Primer extension analysis to detect TIS and P sites was done using 5–10 μg of RNAs and specific 5′end labeled primers, as previously described (Sáez-Vasquez et al., 2004b; Pontvianne et al., 2007). Products of the reaction were analyzed on 8% polyacrylamide/ 7 M urea sequencing gel.

## Author contributions

CM, ND, AO, DP, PC, and CP performed the experiments. MC analyzed data. ND took part in writing the manuscript. FP, CC, and ES supervised experiments. JS conceived, designed the study and wrote the manuscript. All authors approved the final manuscript.

### Conflict of interest statement

The authors declare that the research was conducted in the absence of any commercial or financial relationships that could be construed as a potential conflict of interest. The handling Editor declared a past co-authorship with one of the authors JS, and states that the process met the standards of a fair and objective review.

## Supplementary material

Figure S1 *A. thaliana* nucleolar proteomes. **(A)** Venn diagram of *A. thaliana* of the overlaps of nucleolar proteomes from Palm et al. (2016) (blue), Pendle et al. (2005) (purple) and the present study (green). **(B)** Histogram showing the subcellular distribution of identified nucleolar proteins from *Arabidopsis* leaves according to Palm et al. (2016). Each protein can be localized in only one of the following categories: Cytosol or Nucleus or Nucleus and Cytosol or Nucleolus or Nucleolus and Cytosol or Nucleolus and Nucleus or Nucleolus and Nucleus and Cytosol.

Figure S2 Analysis GO (Gene Ontology) and COG (Cluster of Orthologous Genes) of proteins found in the nucleolus. **(A)** The Table Shows values for the five top GO categories for proteins identified in nucleolar fractions. No, Nucleolar proteins; WCE, Whole Cell Extract proteins. *P*-values and adjusted *p*-values, obtained with a Fisher test, are provided to indicate significant enrichment of specific proteins in the nucleolus compared to proteins detected in WCE. **(B)** The bar graph shows the percentage of each COG categories of proteins identified in the nucleolus. The GO and COG analysis were performed using the website servers http://www.genome.jp/tools/kaas/ and https://www.ncbi.nlm.nih.gov/COG/ respectively.

Figure S3 Subcellular location of 26S proteasome proteins in *A. thaliana* **(A)** Scheme of the 26S proteasome complex showing the 19S Regulatory Particle (RP) and the 20S Core Particle (CP). In blue are represented protein subunits identified in both replicates (exp-1 and exp2) and in orange protein subunits only present in one of the replicate (exp1 or exp-2). In gray are indicated protein subunits non-identified in the nucleolar fractions. **(B)** Immunolocalization of Rpn5a-GFP, GFP-Rpn5a, PBC1/β3-GFP, GFP-PBC1/β3, Rpt5b-GFP, GFP-Rpt5b, and PBG1/β7-GFP and GFP-PBG1/β7 fused proteins in protoplasts of *A. thaliana*. The green signal shows the localization of Rpn5a, PBC1/β3, Rpt5b, and PBG1/β7 proteins. The red signal shows the signal emitted by the nucleolar marker Fibrillarin fused to mCherry (Palm et al., 2016). AUF corresponds to auto fluorescence signal. Scale bar 5 μm.

Figure S4 Immuno-localization of Rpn10 in root tip cells from WT FIB2:YFP plants. Arrows point subnucleolar structures called nucleolar cavities (NoC). Green fluorescence of the Fib2:YFP, is used to visualize nucleolus and DAPI staining to visualize nucleoplasm.

Figure S5 Western blot analysis of nuclear and cytoplasmic protein fractions. Specific antibodies were used to detect nuclear (Histone H3) or cytoplasmic (PRXII) proteins. NUC1 detection serves to verify the absence of NUC 1 protein in *nuc1.2* mutant plants. Membrane was stained with Ponceau-S to verify similar amount of protein in each sample.

Table S1 List of Proteins found in exp-1 nucleoli isolation.

Table S2 List of Proteins found in exp-2 nucleoli isolation.

Table S3 List of Proteins found in both exp-1 and exp-2.

Table S4 Localization of proteins found in both exp-1 and exp-2.

Table S5 List of proteins found in *A. thaliana* whole cell extracts.

Table S6 List of proteins found in exp-1 and exp-2 and belonging to COG O.

Table S7 26S proteasome proteins and accessions in *A. thaliana*.

Table S8 List of oligonucleotides used to clone CDS of Rpn5a, Rpt5b, PBC1/β3, and PBG1/β7 into pRTds vector.
