# Supplementary figures and images for: Nucleolar Proteome Analysis and Proteasomal Activity Assays Reveal a Link between Nucleolus and 26S Proteasome in A. thaliana
Source: Front Plant Sci. 2017 Oct 20;8:1815. doi: 10.3389/fpls.2017.01815 (PMC5655116; doi:10.3389/fpls.2017.01815)

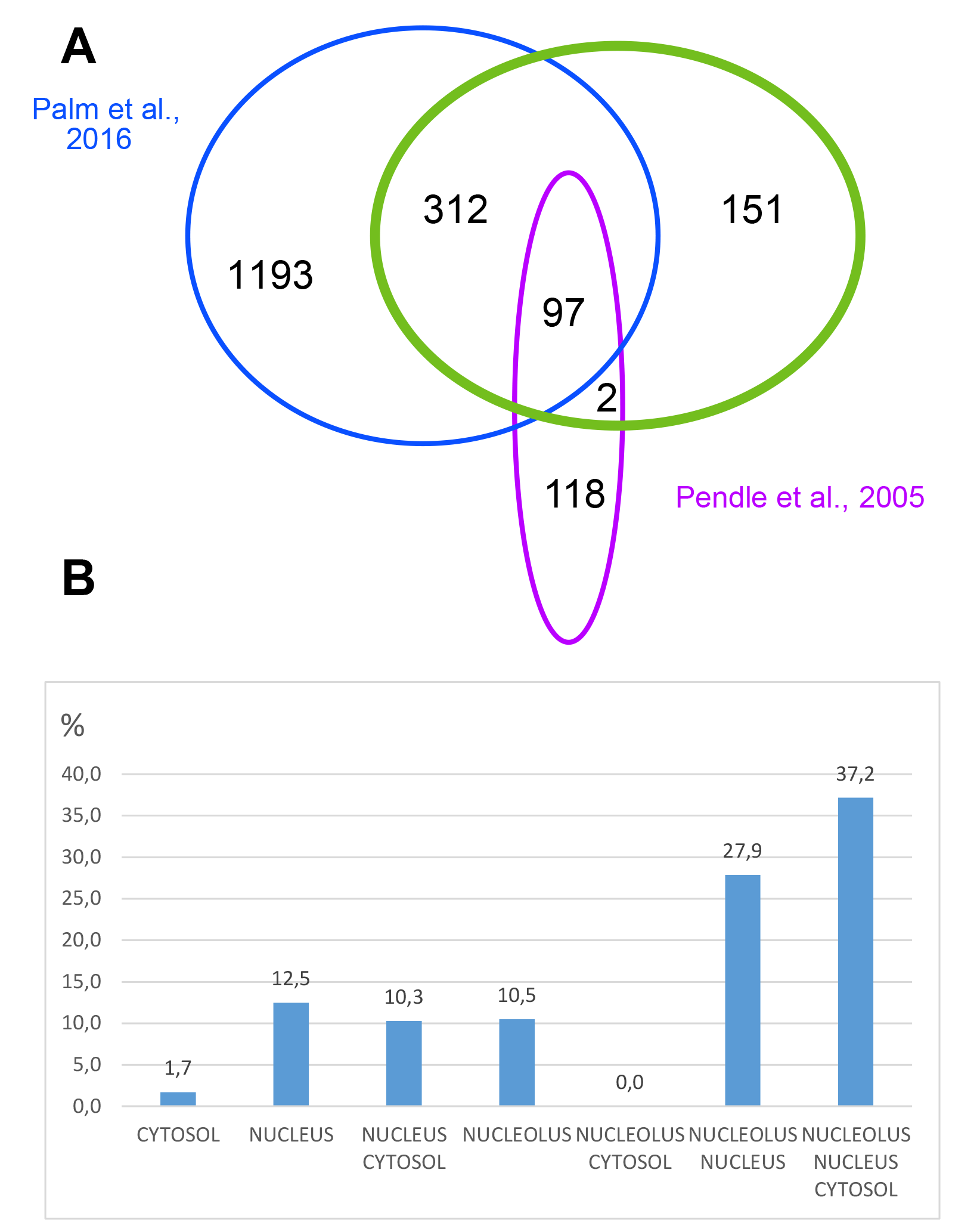

Supplement: Figure S1 — A. thaliana nucleolar proteomes. (A) Venn diagram of A. thaliana of the overlaps of nucleolar proteomes from Palm et al. (2016) (blue), Pendle et al. (2005) (purple) and the present study (green). (B) Histogram showing the subcellular distribution of identified nucleolar proteins from Arabidopsis leaves according to Palm et al. (2016). Each protein can be localized in only one of the following categories: Cytosol or Nucleus or Nucleus and Cytosol or Nucleolus or Nucleolus and Cytosol or Nucleolus and Nucleus or Nucleolus and Nucleus and Cytosol. [file Image1.TIF]

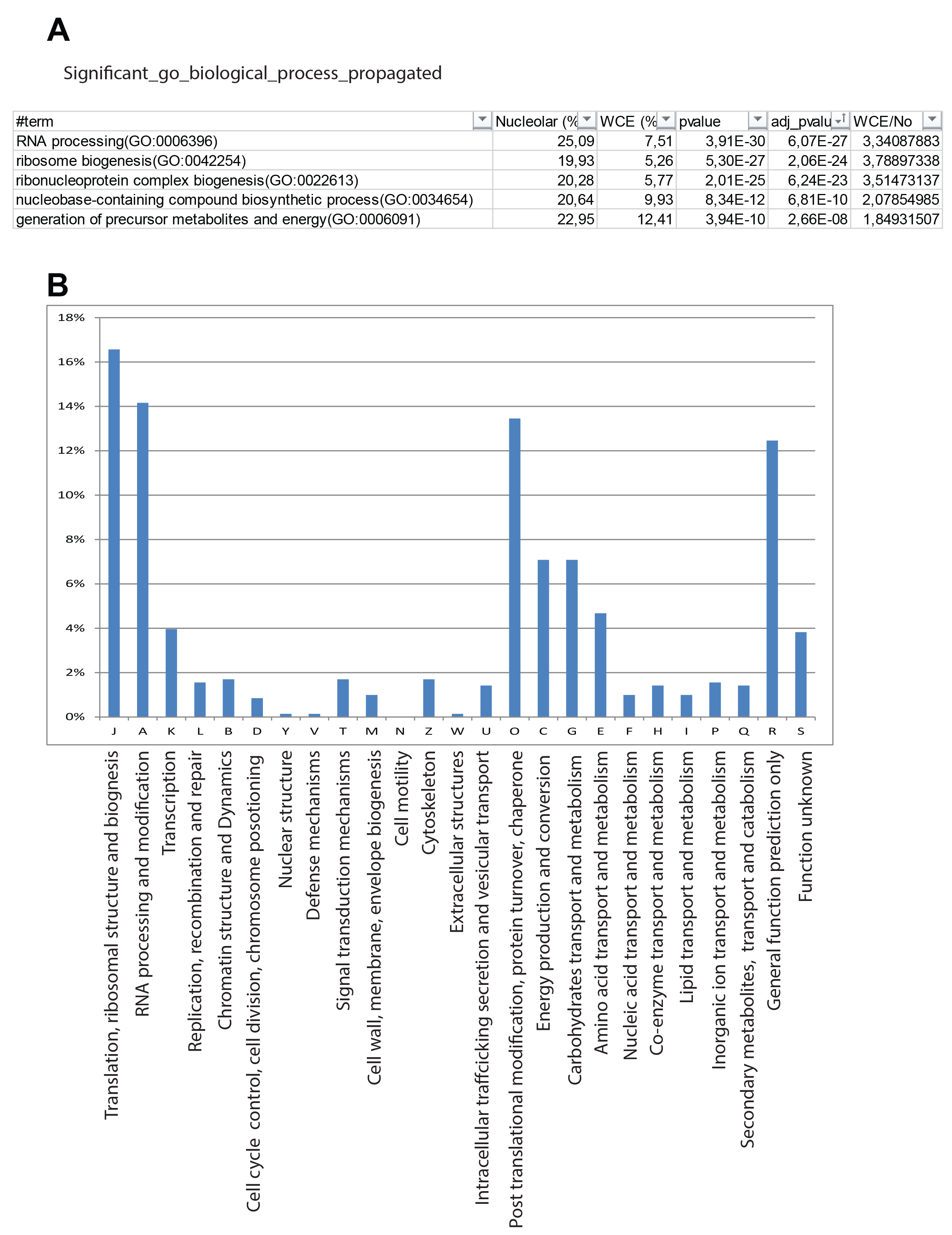

Supplement: Figure S2 — Analysis GO (Gene Ontology) and COG (Cluster of Orthologous Genes) of proteins found in the nucleolus. (A) The Table Shows values for the five top GO categories for proteins identified in nucleolar fractions. No, Nucleolar proteins; WCE, Whole Cell Extract proteins. P-values and adjusted p-values, obtained with a Fisher test, are provided to indicate significant enrichment of specific proteins in the nucleolus compared to proteins detected in WCE. (B) The bar graph shows the percentage of each COG categories of proteins identified in the nucleolus. The GO and COG analysis were performed using the website servers http://www.genome.jp/tools/kaas/ and https://www.ncbi.nlm.nih.gov/COG/ respectively. [file Image2.TIF]

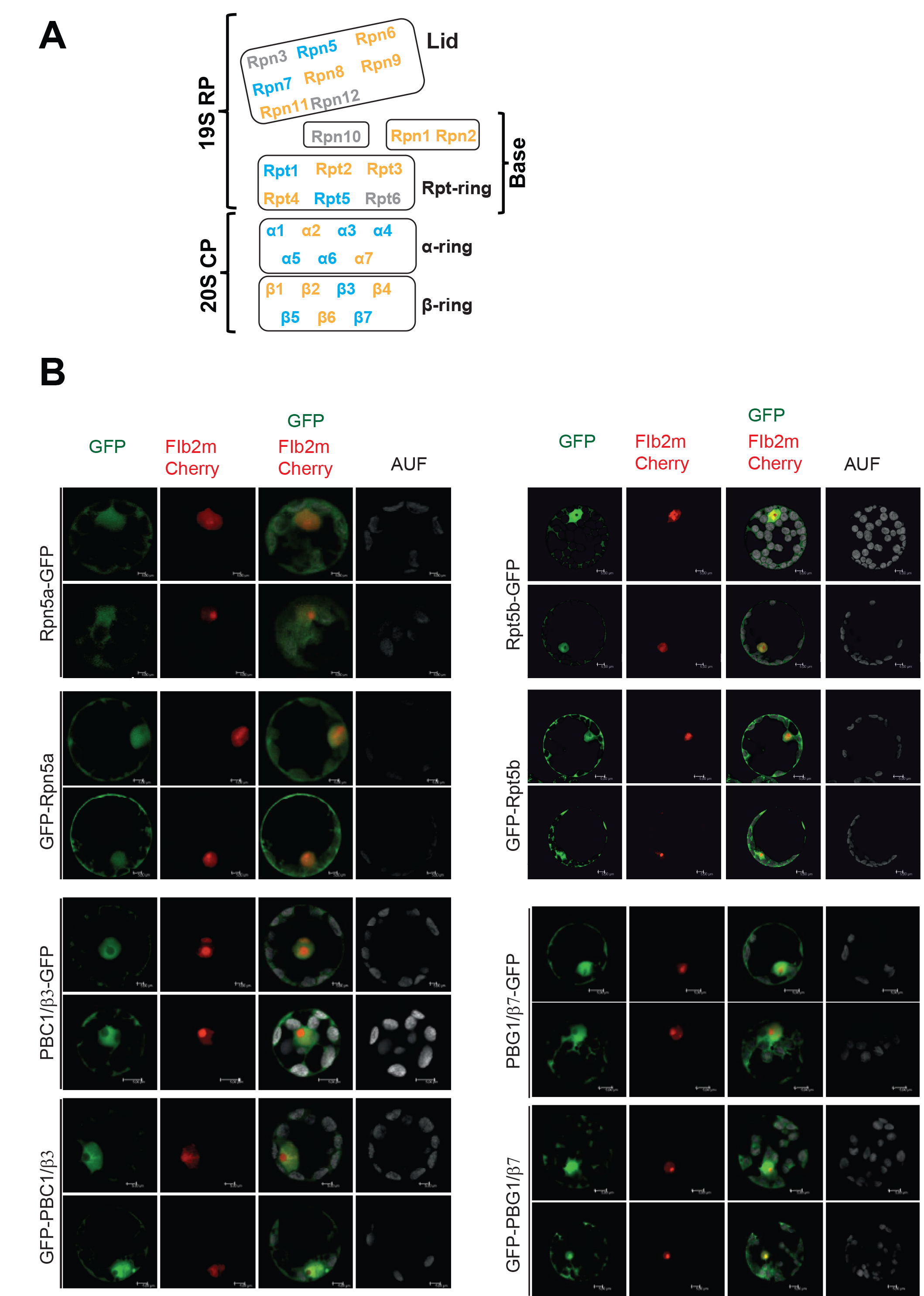

Supplement: Figure S3 — Subcellular location of 26S proteasome proteins in A. thaliana (A) Scheme of the 26S proteasome complex showing the 19S Regulatory Particle (RP) and the 20S Core Particle (CP). In blue are represented protein subunits identified in both replicates (exp-1 and exp2) and in orange protein subunits only present in one of the replicate (exp1 or exp-2). In gray are indicated protein subunits non-identified in the nucleolar fractions. (B) Immunolocalization of Rpn5a-GFP, GFP-Rpn5a, PBC1/β3-GFP, GFP-PBC1/β3, Rpt5b-GFP, GFP-Rpt5b, and PBG1/β7-GFP and GFP-PBG1/β7 fused proteins in protoplasts of A. thaliana. The green signal shows the localization of Rpn5a, PBC1/β3, Rpt5b, and PBG1/β7 proteins. The red signal shows the signal emitted by the nucleolar marker Fibrillarin fused to mCherry (Palm et al., 2016). AUF corresponds to auto fluorescence signal. Scale bar 5 μm. [file Image3.TIF]

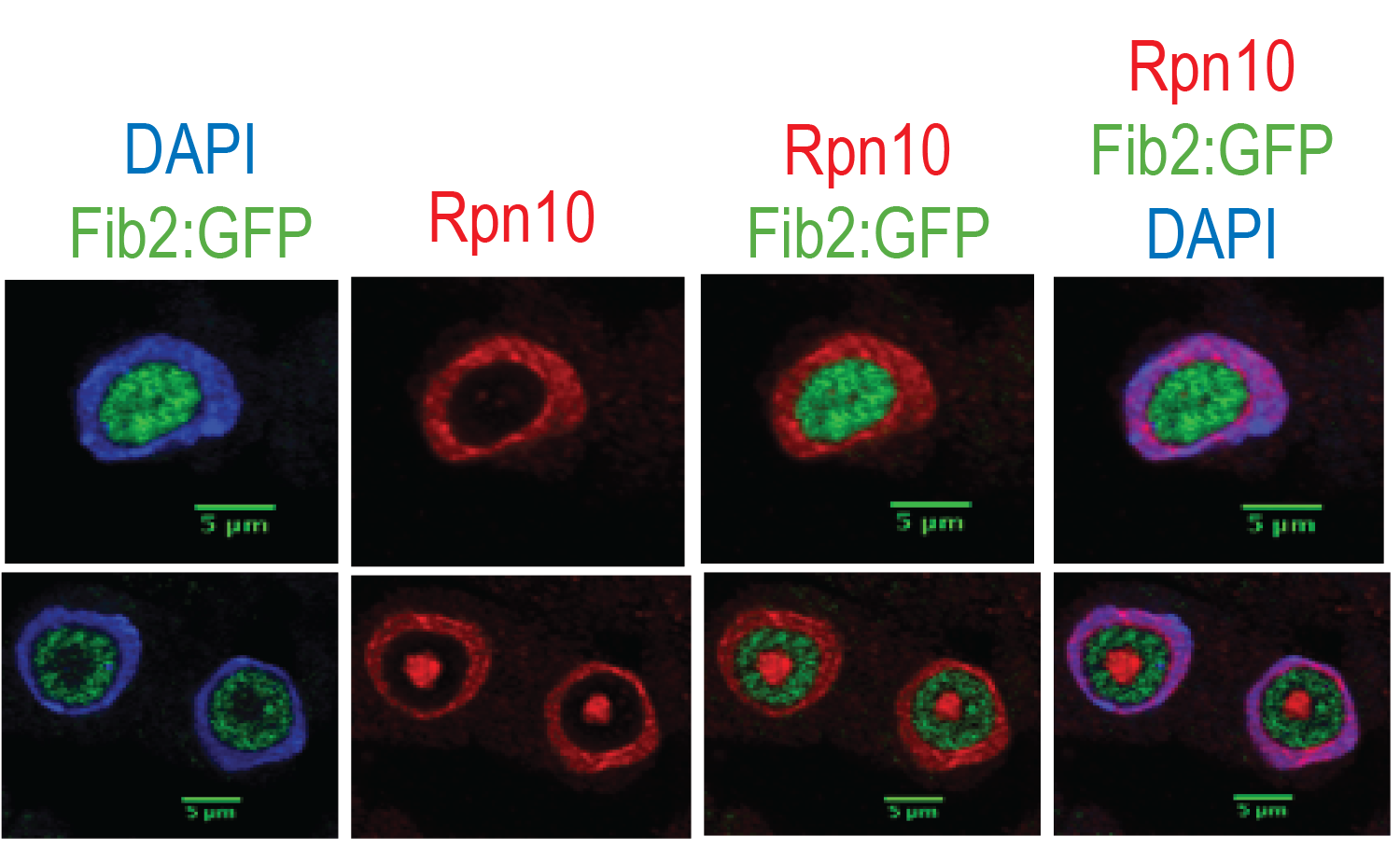

Supplement: Figure S4 — Immuno-localization of Rpn10 in root tip cells from WT FIB2:YFP plants. Arrows point subnucleolar structures called nucleolar cavities (NoC). Green fluorescence of the Fib2:YFP, is used to visualize nucleolus and DAPI staining to visualize nucleoplasm. [file Image4.TIF]

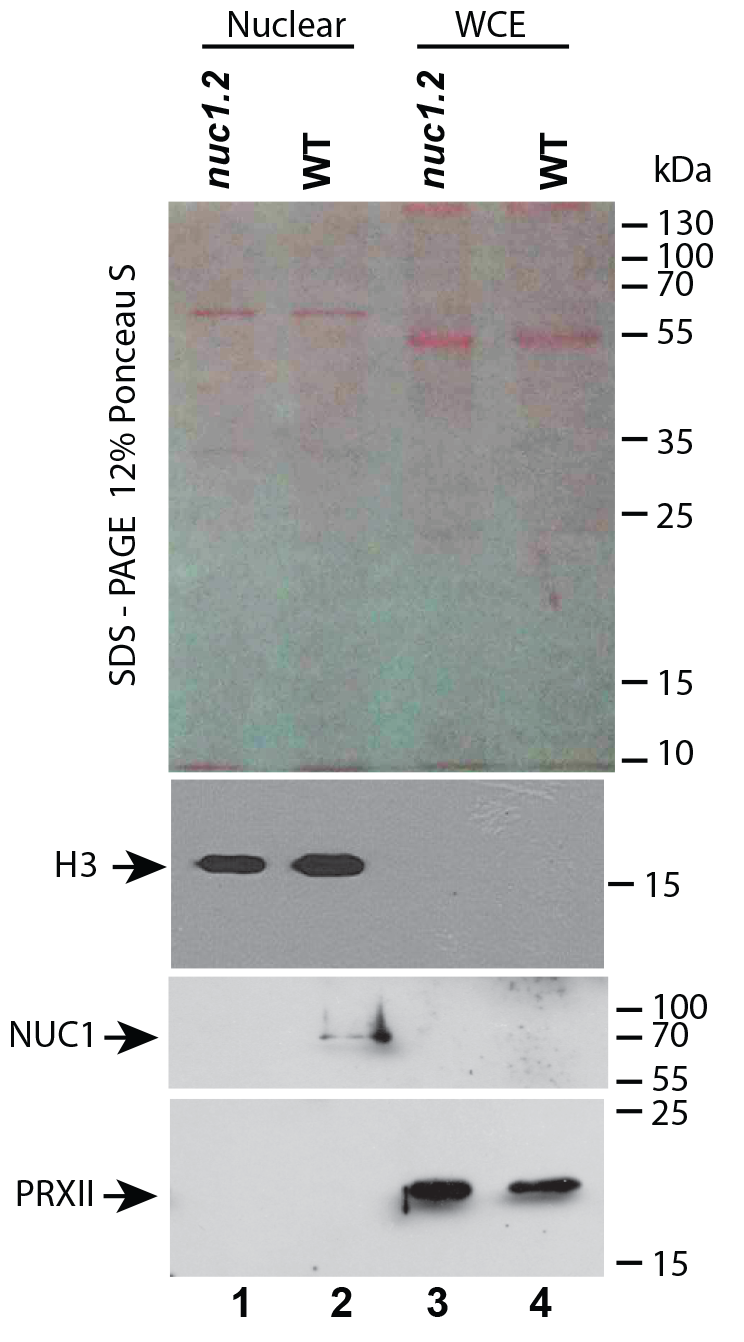

Supplement: Figure S5 — Western blot analysis of nuclear and cytoplasmic protein fractions. Specific antibodies were used to detect nuclear (Histone H3) or cytoplasmic (PRXII) proteins. NUC1 detection serves to verify the absence of NUC 1 protein in nuc1.2 mutant plants. Membrane was stained with Ponceau-S to verify similar amount of protein in each sample. [file Image5.TIF]
